# Mechanistic Differences in Adult-Onset and Juvenile-Onset Huntington’s Disease and the Potential Role of Neurodevelopment

**DOI:** 10.7759/cureus.117175

**Published:** 2026-09-29

**Authors:** Vasundhara Anjur Thupil, Nikhil Satchidanand

**Affiliations:** 1 Neurology, Seven Lakes High School, Katy, USA; 2 Medicine, Ivy Scholars, Houston, USA

**Keywords:** huntington’s disease (hd), juvenile-onset huntington's disease, mutant huntingtin, neurodegeneration, neurodevelopment

## Abstract

Huntington’s disease (HD) is a progressive neurodegenerative disease that primarily affects the striatum and causes cognitive, motor, and psychiatric symptoms. HD onset before the age of 20 is considered juvenile-onset HD (JHD). This review explores the mechanisms and pathology of JHD, with a focus on how ongoing maturation of neural circuits could cause JHD to differ from adult-onset HD (AHD). We identify key mechanistic differences related to the polyglutamine expansion in the *huntingtin* (HTT) gene, transcriptional dysregulation and splicing errors in striatal neurons, dysregulation of synaptogenesis, behaviors of protein aggregates, and failure of proteostasis. We also briefly summarize differences in the disease burden, diagnostic necessities, and treatment options. Overall, there are significant mechanistic and symptomatic differences between AHD and JHD due to the developmental windows during which JHD occurs, and this review highlights the importance of understanding these differences for developing accurate, early diagnostic measures and implementing effective JHD-specific therapies.

## Introduction and background

Huntington’s disease (HD) is a neurodegenerative disease characterized by neuronal degeneration in the striatum, cerebral cortex, and thalamus [1]. HD patients experience a mixture of involuntary movements, behavioral issues, and cognitive impairment [2]. Juvenile-onset HD (JHD) is a form of HD, characterized by symptom onset before or at the age of 20 years [2]. About 5% of HD patients are juvenile-onset [3].

There are significant phenotypic differences between adult-onset HD (AHD) and JHD. While AHD is more commonly considered a hyperkinetic movement disorder, with symptoms including chorea and dystonia, JHD is more commonly considered a hypokinetic movement disorder, with symptoms including rigidity and bradykinesia [4,5]. Additionally, in childhood-onset HD, ongoing brain development significantly influences disease progression, as seen in the symptomatic differences between AHD and JHD [3,6].

HD is caused by an extended cytosine-adenine-guanine (CAG) repeat in exon 1 of the *huntingtin* (HTT) gene, which codes for the production of the HTT protein [7]. Longer CAG repeats are associated with a younger onset and a faster disease progression [2]. Patients with JHD have a much longer CAG repeat than those with AHD [2]. The CAG expansion results in a mutated HTT (mHTT) protein that is associated with disruptions in ordinary cellular functions [8]. Wild-type HTT (wtHTT) is a transcriptional regulator, and a lack of this protein contributes to errors in early brain formation, such as abnormal synaptic pruning [3,9]. The mHTT protein misfolds and aggregates, and toxic buildup of these aggregates can impair functionality, such as blood-brain barrier (BBB) maintenance and protein homeostasis [10,11]. Broader literature suggests that the downstream impacts of this include mitochondrial dysfunction, and the resulting energy deficiency in young neurons can exacerbate failure of mitophagy and worsen oxidative stress [12].

HD is ultimately fatal [13]. The mean life expectancy of AHD after symptom onset is generally between 15 and 20 years [14]. Clinical studies describe substantial variability in disease progression and survival among JHD patients [15-17]. There is currently no way to cure the disease or slow its progression [18]. This review aims to synthesize the pathophysiology of JHD, with a focus on mechanistic differences between AHD and JHD and the influence of neurodevelopment on these differences.

## Review

Methods

Literature Search Strategy

A structured narrative literature review was conducted to identify peer-reviewed publications addressing the epidemiology, pathophysiology, diagnosis, management, and emerging therapeutic approaches for JHD. Electronic literature searches were performed in Google Scholar, PubMed, MEDLINE, and Scopus, as these databases provide comprehensive coverage of biomedical, neurological, and clinical research. Searches were supplemented through manual review of the reference lists of relevant review articles, consensus statements, and landmark publications to identify additional eligible studies. An initial search was completed in November 2024 and updated in August 2026. 

Search Terms

Searches were conducted using combinations of Medical Subject Headings (MeSH) and free-text keywords. Primary search terms included "juvenile Huntington disease," "juvenile Huntington's disease," "juvenile-onset Huntington disease," "pediatric Huntington disease," "childhood-onset Huntington disease," "Westphal variant," "HTT mutation," "CAG repeat expansion," "Huntington disease AND children," and "Huntington disease AND adolescents".

Additional searches combined these terms with epidemiology, clinical presentation, diagnosis, neuropsychiatric, cognitive impairment, motor symptoms, dystonia, rigidity, seizures, neuroimaging, genetics, biomarkers, treatment, multidisciplinary care, prognosis, and disease progression. Boolean operators (AND, OR) were used to combine search terms appropriately.

Study Selection

Articles were screened based on the title and abstract for relevance to the objectives of the review. Full-text articles were subsequently evaluated for inclusion. Priority was given to systematic reviews and meta-analyses; clinical reviews; observational cohort studies; multicenter registry studies; consensus statements; clinical practice recommendations; and seminal publications describing the genetics, phenotype, and natural history of JHD.

Because JHD is a rare disorder, well-documented case series and selected case reports describing unique clinical manifestations or diagnostic challenges were also included when they provided clinically important information.

Eligibility Criteria

Articles were included if they focused primarily on JHD (onset ≤ 20 years of age); addressed clinical, genetic, pathological, diagnostic, or therapeutic aspects of JHD; were published in peer-reviewed journals; and were available in English. Articles published earlier than 1990 were excluded. AHD studies were included only when they provided important comparative information relevant to understanding the distinct features of juvenile disease.

Data Synthesis

Evidence was synthesized narratively rather than quantitatively because of the rarity of JHD, the predominance of observational studies and case series, and the substantial heterogeneity in study design, patient populations, and reported outcomes. Findings were synthesized with particular emphasis on mechanisms relevant to neurodevelopment. A brief overview was included for challenges in diagnosis, treatment opportunities, disease burden, and future research. 

When multiple studies addressed similar topics, greater emphasis was placed on larger cohorts, multicenter investigations, recent consensus reviews, and high-quality systematic reviews while acknowledging evidence derived from smaller observational studies when appropriate. Studies comparing JHD and AHD were prioritized, followed by studies focused on JHD, studies focused on AHD, studies on general mechanisms, and studies on similar mechanisms in similar diseases.

Disease burden

JHD has an estimated minimum incidence of 0.70 per one million patient-years and a minimum estimated prevalence of 6.77 per one million patient-years [19]. Despite JHD’s low incidence, it can profoundly impact patients and caregivers [20]. Relatively few clinicians are aware of JHD’s existence, and the disease often shares symptoms with disorders more commonly observed in children such as autism spectrum disorder, contributing to diagnostic delays that often exacerbate the burden of managing JHD [3,16,17,21]. Parents may also feel emotionally unprepared to accept an HD diagnosis and the realization that they may outlive their children, causing resistance that could further delay proper diagnosis [14,21]. Additionally, lack of understanding can isolate patients, who are entirely dependent on caregivers [16]. It can also isolate caretakers who are often simultaneously caring for multiple family members with HD, must contend with complicated medication regimens, and often report feeling more knowledgeable than clinicians, increasing the burden on them [20-24].

Mechanisms and pathophysiology

Understanding JHD’s underlying mechanisms is critical to developing therapies to reduce disease burden. Additionally, JHD often has a more aggressive pathology, with a different phenotype from AHD, making it crucial to identify mechanistic differences that could provide insights into more effective, early diagnostic and treatment methods [20].

CAG Expansion

Patients with JHD typically have a longer CAG repeat (>60 repeats) in the HTT gene than AHD patients, contributing to earlier disease onset and more aggressive progression [15,24,25]. Symptoms vary by length of CAG expansion, with distinct phenotypes observed in patients with childhood-onset HD, adolescent-onset HD, and JHD [25,26]. Patients with high CAG expansions more commonly experience symptoms such as gait disturbances, developmental delays, and seizures, while patients with low CAG expansions more commonly experience loss of hand dexterity, chorea, and obsessional behavior [25]. However, the increased CAG repeat length does not impair early developmental milestones before symptom onset [27].

The HTT mutation is primarily passed on through autosomal dominant inheritance, though de novo mutations can also occur, and no singular definite function of wtHTT has been identified, though it has been linked to embryogenesis and nervous system development [28-31]. Specifically, the presence of mHTT interferes with many developmental processes such as neuronal maturation, synaptogenesis, and protein development [30,32]. As JHD typically manifests during ongoing development due to the larger CAG expansion, these disruptions may coincide with brain maturation, affecting the disease’s clinical presentation [4,17].

Splicing Errors

HD has also been linked to transcriptional dysregulation by JHD-specific and broader literature, which can negatively impact early neuron development and neurogenesis processes, such as errors in RNA splicing in the striatum in early brain development [33-36].

In HD, splicing regulator PQBP1 abnormally binds to the extended polyglutamine (polyQ) tract in mHTT, and this attraction increases with longer polyQ stretches, which are common in JHD [37]. This binding leads to a deficiency in this regulator, causing many issues, such as striatum underdevelopment [38]. Complete PQBP1 deletion impairs the body’s ability to form spiny projection neurons (SPNs) [38]. In HD, deficiency in this regulator affects medium spiny neurons (MSNs), which are critical for motor control [38]. In JHD, this impacts striatum development, compared to later degeneration in AHD, where MSNs originally function normally [38].

The problems with splicing contribute to the creation of mis-spliced HTT isoforms that can further interfere with striatal functions necessary for motor movements [39,40]. This can trigger premature apoptosis in vulnerable neurons due to interactions with the mutant exon1 HTT fragments, which is likely associated with increased striatal neurodegeneration and aggressive disease progression in JHD [40].

Synaptogenesis Dysregulation

Disruptions to the process of synaptogenesis, seen in dysregulation of dendritic arborization and synaptic pruning, are influenced by mHTT-induced fractalkine reduction [41,42]. This can contribute to the abnormal changes in synaptic pruning seen in HD [43]. Further, brain organoid models have demonstrated impairment of neuronal connectivity and neurite development in HD [44]. Patients with JHD also exhibit striatal hypoconnectivity and disruption of corticostriatal functional connectivity [45]. In JHD, this is seen mostly in the cortico-striato-thalamo-cortical loop and frontostriatal circuit, both of which are considered vulnerable to synaptogenesis disruptions due to lengthy development that sometimes continues into early adulthood [46].

Protein Aggregation Behaviors

JHD is considered a proteinopathy as many of its symptoms can be tied to protein misfolding and aggregation [47]. In JHD, the extended polyQ tract in the produced HTT proteins makes them more likely to misfold and aggregate [48]. Protein aggregation can block important cellular functions from occurring, leading to conditions such as mitochondrial dysfunction and proteasomal impairment [49,50]. When these aggregated proteins create fibrillar structures, they form insoluble amyloids, leading to toxic accumulation in the brain [51]. It is thought that the longer polyQ stretch in JHD leads to faster aggregation, causing more aggressive disease progression [52].

Protein aggregates spread throughout the cells in patients through nucleation and seeding behaviors [53]. In JHD, this behavior becomes more efficient due to the larger polyQ expansion, which is associated with more rapid disease progression compared to AHD [53].

BBB Dysfunction

mHTT aggregates can build up in brain microvascular endothelial cells (BMECs), which play a critical role in the development and maintenance of the BBB, and the following dysregulation of tight junctions increases its permeability [54-56]. This increased permeability may be particularly consequential in JHD because it occurs during ongoing brain maturation, when, as broader studies suggest, the BBB is still essential for maintaining a controlled environment required for normal developmental processes [55].

High permeability of the BBB increases the chances of inflammatory cytokines and peripheral immune cells entering the brain, increasing neuroinflammation, and excessive chronic exposure due to BBB failure can contribute to the development of neuropsychiatric disorders and cognitive impairment [55,57]. The inflammation induced by these peripheral immune cells may be associated with disruptions to development seen in JHD [57].

Additionally, neuroinflammation can worsen developmental vulnerability, disrupting processes that interact with BMECs and the BBB, such as neurogenesis [9]. A potential downstream impact of this is the dysregulation of immune responses [58,59]. The combination of the developing brain in JHD and the weakened BBB is likely associated with greater vulnerability in patients with JHD [60]. Additionally, BMECs regulate blood flow throughout the brain to ensure proper distribution of nutrients and energy, so their dysfunction in JHD is likely associated with the energy deficiencies and mitochondrial dysfunction seen in neurons, which is discussed in more detail later [61].

Proteostasis Failure

Failure of the ubiquitin-proteasome system (UPS) to clear misfolded and aggregated protein fragments contributes to proteostasis dysregulation [62]. This failure occurs in part due to attempts to tag and degrade mHTT fragments, resulting in sequestration of UPS components into mHTT aggregates [63]. In JHD, the longer CAG expansion may result in a greater amount of aggregation-prone mHTT fragments, placing greater strain on the UPS and potentially contributing to earlier or more severe neurodegeneration [62].

One of the main contributing factors to the failure of cellular proteostasis is dysfunction of the autophagy-lysosomal pathway, which is associated with toxic accumulation of mHTT aggregates in the striatum [64,65]. This collapse is thought to be caused in part by mHTT cleavage, which forms N-terminal fragments that are smaller and more prone to aggregation [66]. In JHD, the longer CAG expansion is associated with more rapid cleavage and accumulation of the fragments [66]. Neurons are also more vulnerable during this time due to ongoing brain maturation, meaning this is likely associated with increasing disruptions to neural function in patients with JHD [67].

Loss of protein chaperone networks further weakens cellular defenses against misfolded mHTT, promoting aggregate accumulation [68]. In JHD, this burden is likely disruptive because of ongoing neural progenitor proliferation and differentiation, making developmental processes more vulnerable [69,70].

Mitochondrial Dysfunction

Mitochondria play a key functional role in cells by synthesizing ATP, but they do not function correctly in HD patients [71]. This can create an energy deficit in neurons, which is particularly significant in early JHD cases during ongoing brain development processes that require high amounts of energy [71,72]. This can lead to failure to properly form axonal networks and synapses, which is likely associated with underdevelopment of the brain’s motor circuits [71,72]. The presence of these developments so early in life could be associated with JHD’s more hypokinetic phenotype as compared to the hyperkinetic phenotype of AHD [3]. 

A key result of this is the high prevalence of disruptions to glucose metabolism in HD [73]. The lack of glucose in neurons interrupts the tricarboxylic acid (TCA) cycle, causing neurons to rely on β-oxidation [74]. This is less efficient, which worsens mitochondrial stress and can increase production of reactive oxygen species (ROS) [75]. Reduced activity of TCA cycle enzymes, including α-ketoglutarate dehydrogenase (α-KDGH) and succinate dehydrogenase (SDH), further limits ATP production, contributing to neuronal energy deficits [76].

The process of mitophagy is critical for regulating many processes that fail in HD, including the brain’s failure to maintain energy homeostasis [12,77]. The mitophagy failure occurring in neurons only exacerbates the effects of mitochondrial dysfunction, as the dysfunctional mitochondria are often never removed, worsening the body’s inability to meet the energy needs of the developing brain [78].

Diagnosis

JHD is currently very difficult to diagnose. There are no validated clinical rating scales or assessment tools for children with JHD [18]. Many clinicians currently use the official rating scales for HD, though the phenotypic differences caused by ongoing natural development in patients with JHD reduce the validity of this practice [2,3]. Recently, a modified version of the Unified Huntington’s Disease Rating Scale Total Motor Score (UHDRS-TMS) was assessed in patients with JHD, with the authors noting that the scale’s focus on chorea makes it unreliable in JHD cases where hypokinetic symptoms are much more common [79].

Additionally, JHD has many similar underlying mechanisms to other neurodegenerative and polyQ diseases, leading to struggles in identifying this disease as distinct [79]. The rarity of this disease exacerbates this, as many clinicians may be unaware of the disease or its implications when seen in younger patients [21,22]. Many parents may also be unaware of this disease and may not bring their child to see a clinician immediately after symptoms begin to present [22].

As the symptoms of JHD are similar to those of many other disorders and differ so greatly from those of AHD, the best predictor of JHD in children is family history [79]. However, some ethical considerations arise with early diagnosis due to the nature of this information [79].Some countries do not allow or strongly discourage testing for HD in patients under the age of 18 [80].Additionally, some parents strongly resist diagnosis because they feel overwhelmed and do not wish to know whether their family has the disease [81]. Additionally, conditions such as de novo mutations or genetic anticipation, which occurs when a child presents with JHD earlier than any family members present with signs of HD, can inhibit the effectiveness of this approach [2]. Together, these factors contribute to significant diagnostic delay, with a mean of nine years [2].

Treatment 

Currently, no treatments exist that can cure AHD or JHD, though some are currently undergoing clinical trials. Existing treatments are purely intended for symptomatic relief [81]. However, even among these treatments, the significant mechanistic and symptomatic differences between AHD and JHD mean that medications do not easily transfer, as JHD medications must additionally consider pediatric medical constraints [80]. For example, antidepressant use is less common in JHD than AHD, potentially due to concerns over the negative impacts of these medications in children [82]. 

Additionally, several symptoms are more common in JHD than AHD, such as seizures, which prevent AHD treatment regimens from being entirely generalizable to patients with JHD as well [83,84]. For example, the most common category of medication in AHD is chorea treatment, but in JHD, the more hypokinetic symptoms such as rigidity and bradykinesia are often worsened by medications intended to treat chorea [16,17,21,85]. Similarly, antipsychotic medications, which are commonly used in AHD, often have Parkinsonian side effects, which can exacerbate existing disease symptoms in patients with JHD [86]. Anti-Parkinsonian medications are also frequently prescribed due to hypokinetic symptoms, but this raises concerns about whether medications are being prescribed for real symptoms or side effects of other treatments [87].

Various nonpharmacological treatment options have been evaluated in HD patients, including dietary interventions, but the use of nutritional therapies poses many concerns in adolescents and teenagers, the effects of which would likely be exacerbated when paired with JHD’s neurodevelopmental pathology [88,89].

Future research

The main direction for future research into JHD is to more clearly determine the differences between AHD and JHD to elucidate differences in future treatment options. While no widespread cures for HD currently exist, various factors must be considered in the development of current and future treatments. For example, an important consideration is the impact of larger CAG repeats on the structure of mHTT and its interactions, which could affect the efficacy of medications intended to lower mHTT levels by changing the characteristics of their target.

Another crucial factor is the importance of wtHTT in many processes essential for homeostasis [31]. Because of this, a treatment must selectively target only the mutated versions of the protein. However, the implications of allele-selective drugs, which are typically used to achieve this purpose, are not well understood in children, such as the impacts on the developing brain. Though this selective targeting of only mHTT has successfully been achieved in mice, the long-term impacts of a reduced level of wtHTT have not been determined [90]. We expect that this would be exacerbated in patients with JHD when going through important developmental milestones such as puberty.

In addition, there is a lack of understanding about the exact timing of when aggregation begins due to significant differences even among patients with JHD ​​​​​​​[25]. Understanding this timing could influence optimal treatment windows, which could allow for more efficient treatment of the disease while minimizing side effects.

## Conclusions

JHD is likely a more complex condition than simply the earlier appearance of symptoms and processes seen in AHD. As symptoms emerge during ongoing development, this timing may influence disease progression, underlying pathophysiology, and clinical presentation. The interactions between disruptions to development and neurodegeneration could contribute to some of the differences between JHD and AHD, although the exact impact of these interactions remains unknown.

Increased awareness of the differences between JHD and AHD could support earlier identification and more individualized management. However, these conclusions are limited by the rarity of JHD, inconsistent definitions, small study populations, and frequent extrapolation of AHD findings to patients with JHD. Further research is needed to determine how developmental stage, CAG repeat length, and other factors interact to influence disease expression and guide treatments to better address the needs of patients with JHD and their caregivers. In addition, because direct mechanistic studies in JHD are limited, several of the proposed links between neurodevelopmental processes and the JHD phenotype remain biologically plausible hypotheses derived from converging evidence rather than mechanisms demonstrated specifically in JHD models. As this was a narrative review, formal risk-of-bias or methodological quality assessments were not conducted, and data were not collected on the number of articles that underwent full-text assessment for inclusion.
